# Characterization of mAbs against *Klebsiella pneumoniae* type 3 fimbriae isolated in a target-independent phage display campaign

**DOI:** 10.1128/spectrum.00400-24

**Published:** 2024-06-28

**Authors:** Sophia K. Berry, Steven Rust, Lorraine Irving, Josefin Bartholdson Scott, Lucy A. Weinert, Gordon Dougan, Graham Christie, Paul Warrener, Ralph Minter, Andrew J. Grant

**Affiliations:** 1 Department of Veterinary Medicine, University of Cambridge, Cambridge, United Kingdom; 2 Antibody Discovery and Protein Engineering, Biopharmaceuticals R&D, AstraZeneca, Cambridge, United Kingdom; 3 Cambridge Institute for Therapeutic Immunology & Infectious Disease, Department of Medicine, University of Cambridge, Cambridge, United Kingdom; 4 Department of Chemical Engineering and Biotechnology, University of Cambridge, Cambridge, United Kingdom; 5 Microbial Sciences, Biopharmaceuticals R&D, AstraZeneca, Gaithersburg, Maryland, USA; Suranaree University of Technology, Nakhon Ratchasima, Thailand

**Keywords:** *Klebsiella pneumoniae*, scFv, monoclonal antibody, MrkA, type 3 fimbriae

## Abstract

We used phage display, antibody engineering, and high-throughput assays to identify antibody-accessible targets of *Klebsiella pneumoniae*. We report the discovery of monoclonal antibodies (mAbs) binding to type 3 fimbrial proteins, including MrkA. We found that anti-MrkA mAbs were cross-reactive to a diverse panel of *K. pneumoniae* clinical isolates, representing different O-serotypes. mAbs binding to MrkA have previously been described and have been shown to provide prophylactic protection, although only modest protection when dosed therapeutically *in vivo* in a murine lung infection model. Here, we used a combination of binding and opsonophagocytic killing studies using a high-content imaging platform to provide a possible explanation for the modest therapeutic efficacy *in vivo* reported in that model. Our work shows that expression of *K. pneumoniae* type 3 fimbriae in *in vitro* culture is not homogenous within a bacterial population. Instead, sub-populations of bacteria that do, and do not, express type 3 fimbriae exist. In a high-content opsonophagocytic killing assay, we showed that MrkA-targeting antibodies initially promote killing by macrophages; however, over time, this effect is diminished. We hypothesize the reason for this is that bacteria not expressing MrkA can evade opsonophagocytosis. Our data support the fact that MrkA is a conserved, immunodominant protein that is antibody accessible on the surface of *K. pneumoniae* and suggest that additional studies should evaluate the potential of using anti-MrkA antibodies in different stages of *K. pneumoniae* infection (different sites in the body) as well as against *K. pneumoniae* biofilms in the body during infection and associated with medical devices.

**IMPORTANCE:**

There is an unmet, urgent need for the development of novel antimicrobial therapies for the treatment of *Klebsiella pneumoniae* infections. We describe the use of phage display, antibody engineering, and high-throughput assays to identify antibody-accessible targets of *K. pneumoniae*. We discovered monoclonal antibodies (mAbs) binding to the type 3 fimbrial protein MrkA. The anti-MrkA mAbs were found to be highly cross-reactive, binding to all *K. pneumoniae* strains tested from a diverse panel of clinical isolates, and were active in an opsonophagocytic killing assay at pM concentrations. MrkA is important for biofilm formation; thus, our data support further exploration of the use of anti-MrkA antibodies for preventing and/or controlling *K. pneumoniae* in biofilms and during infection.

## INTRODUCTION

*Klebsiella pneumoniae* is the leading cause of multi-drug-resistant infections and a significant global health problem (1). *K. pneumoniae* is found ubiquitously in nature and is also found in the healthcare environment on surfaces and on medical devices (2, 3). *K. pneumoniae* is a commensal organism in mammals, colonizing the gastrointestinal tract and nasopharynx in a benign manner, but can disseminate to other tissues and cause severe infections of the urinary tract, lung, bloodstream, and wound sites (2, 4). In developing countries, it is the most common cause of neonatal sepsis, accounting for 16%–28% of cases (5). Mortality rates for *K. pneumoniae* neonatal sepsis are high (66.6%) in comparison to neonatal sepsis caused by other bacteria (21.5%) (6). The dissemination of extended spectrum β-lactamases and *K. pneumoniae* carbapenemases is concerning (7). The mortality rate for carbapenem-susceptible *K. pneumoniae* infections is ~21%, and for carbapenem-resistant infections, it is 41% (8). For more information, many excellent publications describe the epidemiology of *K. pneumoniae* (9–15).

Increasing antimicrobial resistance (AMR) has made *K. pneumoniae* infections challenging to treat. The dissemination of untreatable *K. pneumoniae* infection spreading through hospital-acquired infections, as well as in communities, is a real threat. With some strains now resistant to all classes of antimicrobials recommended to treat the infection, coupled with the current lack of a protective vaccine, *K. pneumoniae* has been included on the WHO list of “high-priority pathogens” for which new therapeutics are urgently needed (1, 4, 16, 17).

Monoclonal antibodies (mAbs) offer an alternative to classical antimicrobials and are the fastest growing class of therapeutics (18). Antimicrobial antibodies usually work by either binding to and neutralizing bacterial virulence mechanisms, or by binding to bacteria and subsequently promoting the activation of the complement system and/or the recruitment of phagocytic cells by Fc receptors (19); however, antimicrobial antibodies with direct bactericidal activity have also been reported (20, 21). Several antibacterial mAbs targeting *K. pneumoniae*, *Staphylococcus aureus*, *Pseudomonas aeruginosa,* and other ESKAPE pathogens are currently in clinical or pre-clinical trials (22–26).

One approach for the generation of therapeutic mAbs utilizes diverse combinatorial libraries of antibody heavy and light chain variable domains (*V*_*H*_ and *V*_*L*_), using phage display technology *in vitro* (27). These single-chain variable fragments (scFvs) have a single antigen-binding site, comprising fusing *V*_*H*_ and *V*_*L*_ through a flexible polypeptide linker (28). Antibody discovery campaigns targeting *K. pneumoniae* have been described, for example, target-directed approaches toward surface polysaccharides such as lipopolysaccharide (LPS) and capsular polysaccharide (CPS) (29–35). There are more than 80 different *K. pneumoniae* capsule serotypes for which no single type predominates, making vaccines and interventions against CPS difficult (1). There are only eight recognized *K. pneumoniae* O serotypes of which four (O1, O2a, O3, and O5) account for most human infections globally, with O1 being dominant in human disease (16, 36–40). A recent, large, unselected, 10-year sequencing-based serotype analysis of *K. pneumoniae*-associated bloodstream infections and available global sequences strongly supports the development of effective O-antigen-targeted approaches to reduce morbidity, mortality, bloodstream infections, and AMR associated with *K. pneumoniae* (16). Several mAbs targeting O-antigen of the LPS have been reported spanning O1, O2, O3, and O5 serotypes (29, 30, 41, 42).

Recently, we, and others, have reported target-independent phage display approaches using whole *K. pneumoniae* bacteria. One study utilized wild-type (WT) *K. pneumoniae* 43816 and a CPS/O-antigen-deficient double-mutant *K. pneumoniae* 43816 Δ*cpsBwaaL*, and identified mAbs targeting the major type 3 fimbrial (T3F) subunit MrkA (43). The mAbs were isolated after three rounds of phage display selections and were shown to promote opsonophagocytic killing (OPK) activity and reduce lung burden in a murine model of pneumonia.

Another target-independent screen reported recently was an independent study utilizing *K. pneumoniae* 43816, a CPS-deficient mutant (*K. pneumoniae* 43816 Δ*cps*), and a CPS/O-antigen-deficient double mutant (as used in the study highlighted above). We recently reported on one mAb, named B39, that exhibited dual binding to both O1 and O2 *K. pneumoniae* strains (41). The mAb promoted potent OPK activity and protected mice when dosed therapeutically in lethal models of O1 and O2 pneumonia (41). Yet, even with the limited number of O serotypes, a cocktail of serotype-specific mAbs may be required for broad strain coverage and protection, or rapid accurate diagnosis may be required prior to administration, or targeting additional antigens may be necessary (26, 40, 43).

Target-independent screening has been used successfully in antibody discovery (23, 41, 43) and is not reliant upon target-dependent hypotheses, which may or may not result in the desired functional effect. Instead, large antibody repertoires can be isolated against a diverse cell-surface-accessible target landscape and then assessed for the desired functional activity. This approach is focused on function and has the possibility of discovering novel biology as well as molecular targets. In our previous study (41), we reported on carbohydrate-targeting mAbs. However, within our phage display campaign, mAbs targeting proteinaceous epitopes were also found. We hypothesized that protein targets may be highly conserved and might offer broad strain coverage and protection. Therefore, this study aimed to explore the protein-targeting mAb repertoires generated previously to isolate broadly reactive mAbs targeting *K. pneumoniae* surface proteins.

## RESULTS

### Primary phage display campaign

**Fig 1:**
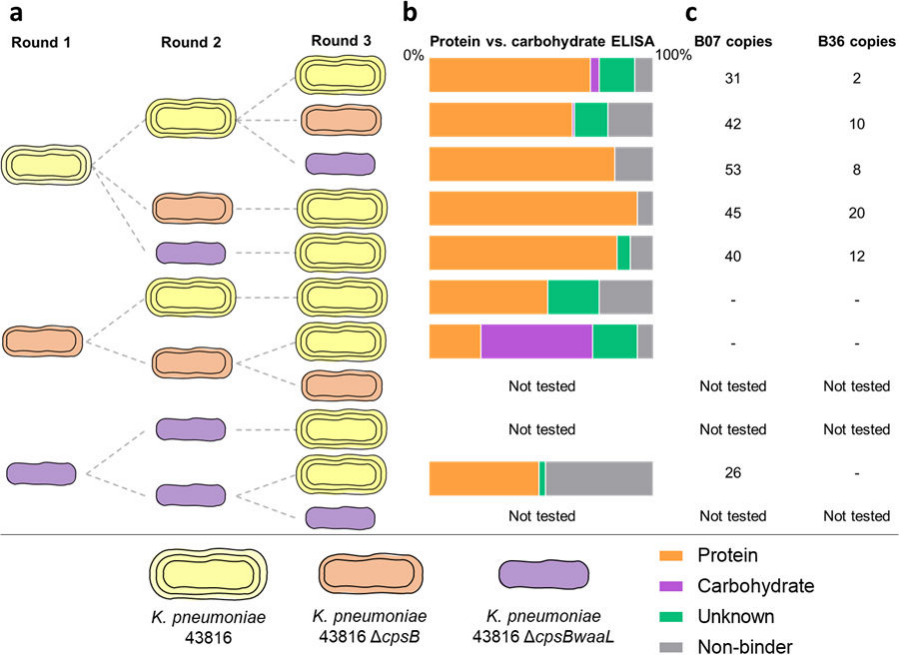
Overview of the antibody discovery campaign. (**a**) Flow diagram shows the *K. pneumoniae* strains used in each round of the phage display selection campaign. Dashed lines indicate the origin of the input library. (**b**) The proportion of protein, carbohydrate, unknown, and non-binders is shown for each round 3 population tested. (**c**) The copy number (out of 88) of the highly prevalent mAbs B07 and B36 is shown for each round 3 population.

As described previously (41), we utilized an scFv phage library to screen live *K. pneumoniae* with the original aim of isolating therapeutic mAbs to treat *K. pneumoniae* infections. The *K. pneumoniae* bacterial cell surface is covered in a layer of polysaccharide comprising CPS, a thick layer of densely packed fibers comprising complex acidic polysaccharides and uronic acids (44), and O-antigen, comprising repeating carbohydrate subunits that extend from the outer core region of the LPS. We hypothesized that these prominent *K. pneumoniae* surface antigens could hinder the phage display process by acting as a sink for scFvs or by shielding surface antigens during phage display. Therefore, in our phage display campaign, we utilized a wild-type strain (*K. pneumoniae* 43816), a mutant deficient in CPS (*K. pneumoniae* 43816 Δ*cpsB*), and a mutant lacking CPS and O-antigen surface expression (*K. pneumoniae* 43816 Δ*cpsBwaaL*). The extensive phage display campaign was described previously (41); an overview is summarized in Fig. 1a.

Anti-*K*. *pneumoniae* antibodies were enriched over three rounds of selection for each of the three bacterial strains. Additional selections were also performed in which the target strain was changed between rounds, for example, performing two rounds of enrichment against *K. pneumoniae* 43816 Δ*cpsBwaaL* and a third round against *K. pneumoniae* 43816. Forty-four antibodies per round 3 population were sequenced to determine the *V*_*H*_ and *V*_*L*_ diversity and tested for binding to *K. pneumoniae* 43816 WT and bovine serum albumin (BSA) by phage ELISA to determine specific and non-specific binders. Poor quality round 3 populations that exhibited a low proportion of specific binders, a high proportion of non-specific binders, and/or a low complementarity-determining region 3 diversity (%) were eliminated at this stage.

### Target exploration

To understand the target landscape of our antibody populations, 88 antibodies per population were screened in a protein vs carbohydrate phage ELISA. Most of the antibody populations were dominated by protein binders, while carbohydrate-targeting scFvs were generally rare (Fig. 1b). The exception to this was an antibody population that was enriched using *K. pneumoniae* 43816 Δ*cpsB* in the first and second rounds of selection and *K. pneumoniae* 43816 in the third round. mAbs originating from this output have been characterized and were shown to bind to the O-antigen of the LPS (41).

**Fig 2:**
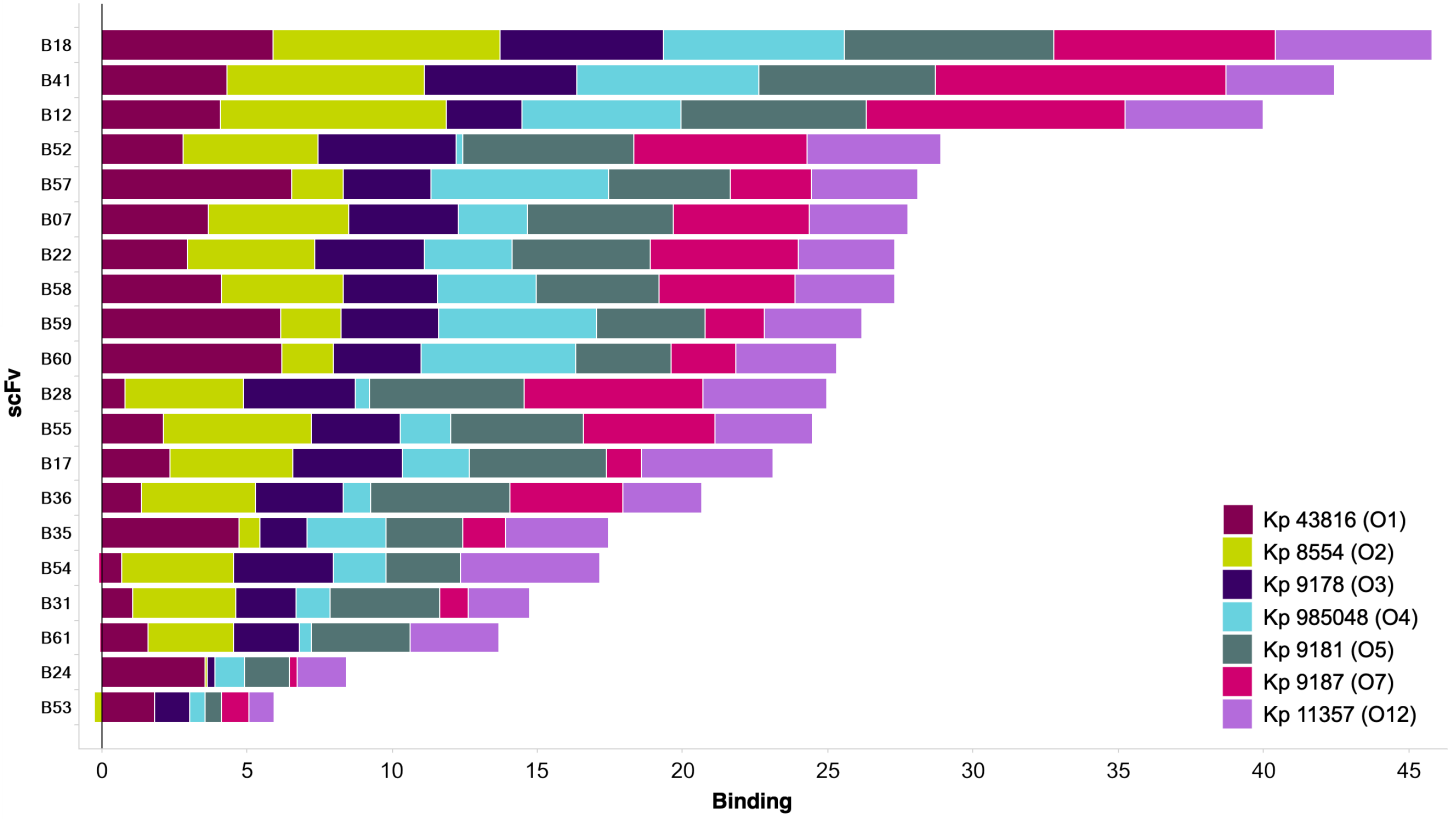
Cross-reactivity of protein-targeting scFv. Each bar represents a unique scFv, and colors represent binding to each *K. pneumoniae* strain. Only the top 20 unique scFvs are shown. Strains used and their O-type are listed. Binding data represent fold-change over an isotype control.

With the aim of identifying mAbs with broad specificity to *K. pneumoniae* proteinaceous antigens, we interrogated the remaining mAbs that bound to proteins. Analysis of *V*_*H*_ and *V*_*L*_ sequences of the protein-targeting antibodies revealed several highly dominant mAbs, including B07 that was represented 237 times across six of the eight outputs tested, and B36 that was represented 52 times across five outputs (Fig. 1c). We next performed a phage ELISA using a panel of representative *K. pneumoniae* strains to screen protein-targeting scFvs for cross-reactivity. Using a maximum likelihood phylogenetic tree and information on O-antigen serotype, the following 6 representative strains were selected from a panel of 31 clinical isolates for cross-reactivity assays: *K. pneumoniae* 8554 (O2), *K. pneumoniae* 9178 (O3), *K. pneumoniae* 985048 (O4), *K. pneumoniae* 9181 (O5), *K. pneumoniae* 9187 (O7), and *K. pneumoniae* 11357 (O12) (Fig. S1). The parent strain *K. pneumoniae* 43816 (O1) was also included in the panel. Several scFvs were identified that bound to all seven strains by phage ELISA (Fig. 2), suggesting that scFv targeting conserved proteinaceous antigens had been enriched for in the phage display campaign. The top 20 were converted to the bivalent scFv-Fc format for further target identification and characterization.

**Fig 3:**
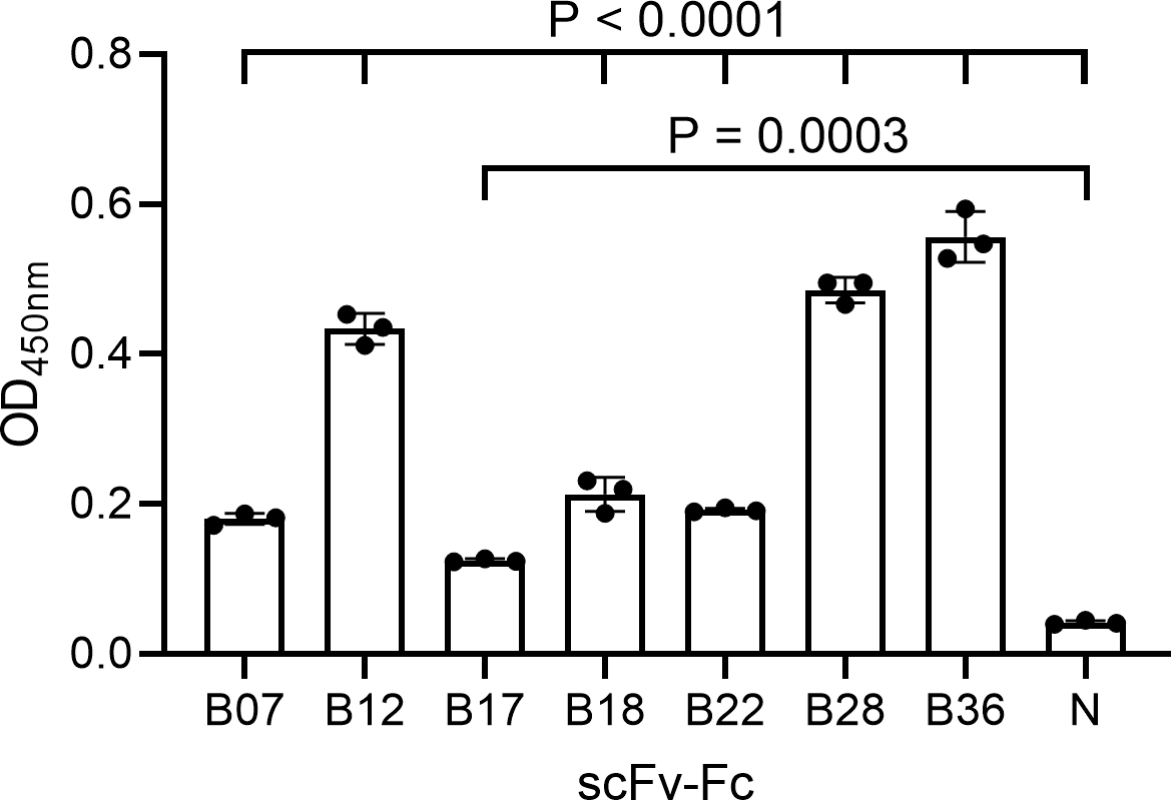
Binding of scFv-Fc to recombinant MrkA by ELISA. Error bars represent 1 SD. *N* = negative control scFv-Fc. *P*-values from a one-way ANOVA are shown above.

In a previous mAb discovery campaign targeting *K. pneumoniae* 43816, both a phage display and hybridoma approach resulted in mAbs binding to the type 3 fimbrial subunit MrkA (43). Therefore, we tested mAbs at 5 µg/mL for binding to recombinant MrkA by ELISA. Seven of the protein-targeting mAbs were found to bind recombinant MrkA (Fig. 3). Of the mAbs that bound to MrkA by ELISA, B12, B28, and B36 exhibited high levels of binding (>0.6 OD_450nm_), while B07, B17, B18, and B22 displayed lower levels of binding (around 0.2 OD_450nm_). We postulated that the different levels of binding could be due to differing target epitopes which could result in differing functional characteristics. As such we decided to select one mAb from each binding level group for further characterization. Due to the clonal dominance exhibited by B07, a medium binder, and B36, a high binder, in the phage display campaign, these scFvs were converted to IgG for further investigation.

### Functional characterization of mAbs-targeting MrkA

**Fig 4:**
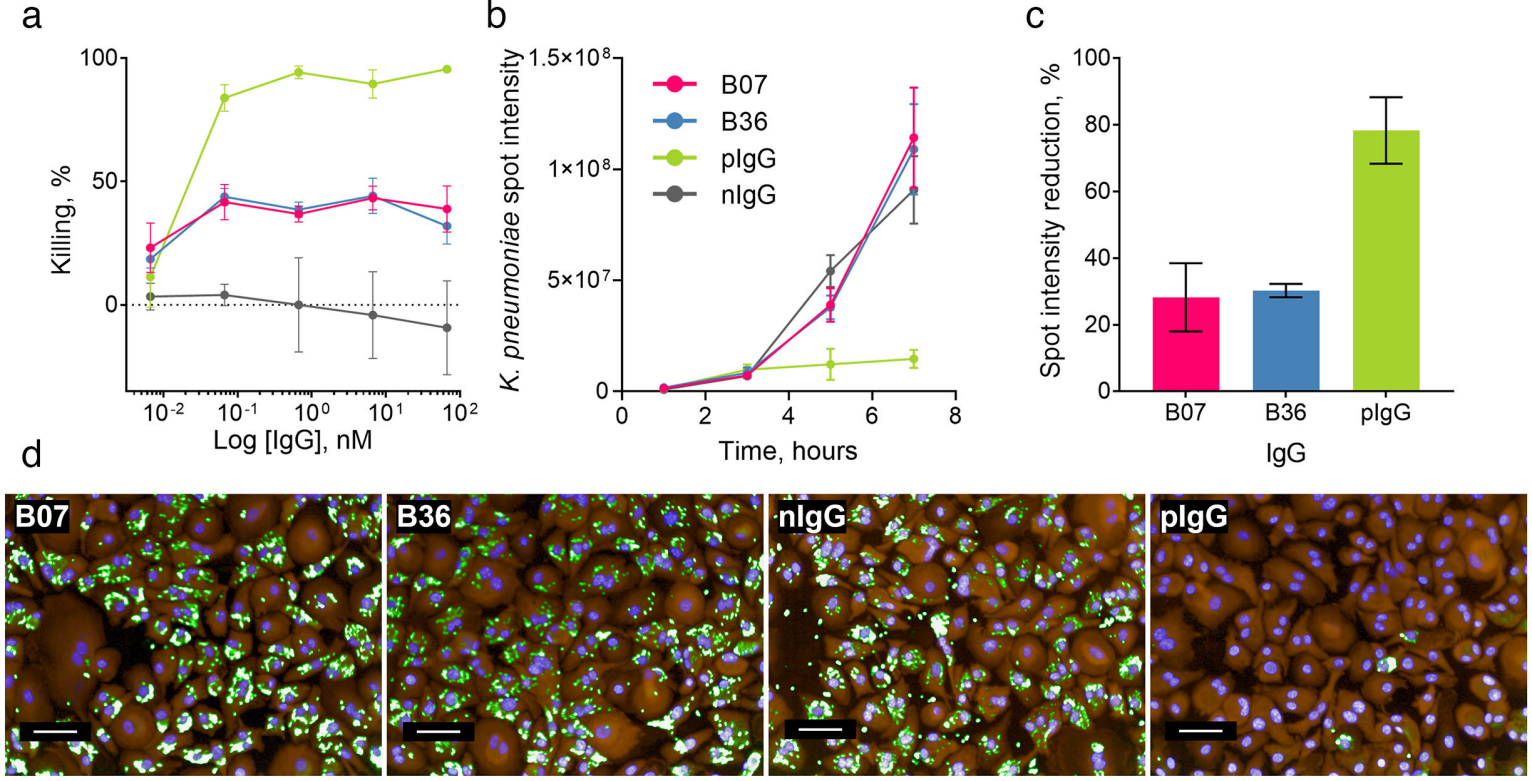
Opsonophagocytic killing of *K. pneumoniae* 43816 Δ*cpsB* by macrophages in the presence of MrkA-targeting mAbs. (a) *K. pneumoniae* 43816 Δ*cpsB lux* bacteria, mAbs, and complement were added to plates containing macrophages and incubated for 5 hours. Luminescence was measured using an Envision multilabel plate reader (PerkinElmer). pIgG, O-antigen-binding mAb (green); nIgG, negative isotype control (gray). Killing by test mAbs or control mAb was calculated as a percentage of wells containing no mAb using the following calculation: (mAb treatment/no mAb) × 100. Error bars represent 1 SD. *N* = 3 individual macrophage donors. For (b), (c), and (d), fixed and permeabilized macrophages were treated with rabbit polyclonal anti-*K*. *pneumoniae* 43816 and stained with the nuclear stain Hoechst (blue), macrophage stain cell mask orange (orange), and AF488 anti-rabbit IgG (green). Images from 15 fields were acquired using an Operetta system at 20× magnification. Scale bar represents 50 µm. (b) Quantification of AF488 intensity sum of *K. pneumoniae* spots in the macrophage cytoplasmic region. (c) Reduction in *K. pneumoniae* spot intensity at 5 hours compared to the control mAb. (d) Representative images of bacterial clearance after a 7-hour incubation. Note: This experiment was performed alongside testing mAbs reported in a previous publication by Berry et al. (41); the control images and data shown in this figure correspond to Nip223 (nIgG) and B39 (pIgG) in Berry et al. (41).

We performed an OPK assay using human monocyte-derived macrophages (MDMs) to identify mAbs with potential therapeutic activity. Due to the anti-phagocytic nature of CPS, both *K. pneumoniae* 43816 and *K. pneumoniae* 43816 Δ*cpsB* strains were tested. We utilized strains harboring a plasmid containing the *luxABCDE* operon, allowing a luminescence-based readout (29, 30, 43). We compared the MrkA-targeting mAbs to a positive control IgG that binds to the *K. pneumoniae* O-antigen and exhibits potent OPK activity which we described previously (41). At concentrations of 66.67 pM or higher, both B07 and B36 promoted a 45% reduction in luciferase signal compared to the isotype control, indicating a reduction in the total number of bacteria per well. At the lowest concentration tested, both mAbs promoted around 20% killing (Fig. 4a). In line with previous reports, no activity was observed against encapsulated bacteria (41, 43, 45) (Fig. S2).

Next, we visualized macrophage-associated bacteria following opsonophagocytosis over a 7-hour time course using high-content imaging (HCI). At 5 hours, treatment with MrkA-targeting mAbs promoted a 30% reduction in macrophage-associated *K. pneumoniae* 43816 Δ*cpsB* spot intensity in comparison to the negative control mAb (Fig. 4c), a result in line with the luminescence-based OPK assay. However, at 7 hours, while enhanced clearance of *K. pneumoniae* by the O-antigen targeting positive control mAb was retained, activity was lost with treatment by the MrkA-targeting mAbs (Fig. 4b and d).

### High-content imaging of MrkA-targeting mAbs binding to *K. pneumoniae*

**Fig 5:**
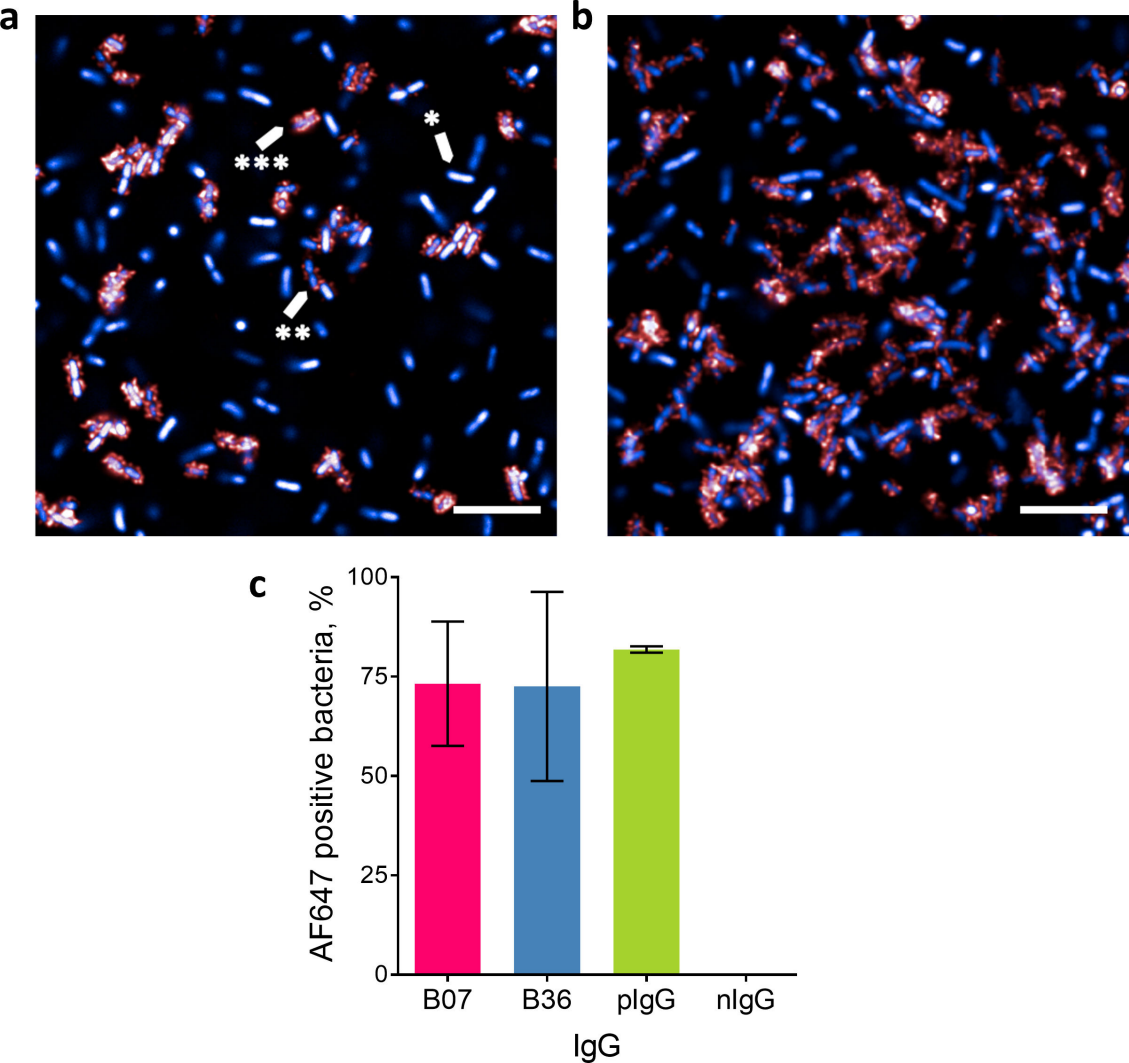
Binding of MrkA-targeting mAbs to *K. pneumoniae* 43816 WT by HCI. (**a**) and (**b**) Representative images of B07 binding to bacteria. Due to variation observed between replicates, two replicates are shown where the proportion of the bacterial population with MrkA staining was lower (**a**) or higher (**b**). Labeled arrows indicate bacteria heavily decorated in MrkA (***), bacteria with some decoration (**), and bacteria with no decoration (*). Scale bar represents 10 µm. (**c**) Proportion of bacteria expressing MrkA when probed with B07, B36, or control mAbs. AF647-positive bacteria (%) represent the percentage of bacteria with a shell region AF647 intensity >3,000 divided by the total number of bacteria. pIgG, O-antigen-binding mAb; nIgG, negative isotype control. Fixed bacteria were treated with mAbs at 1 µg/mL and then stained with 4′,6-diamidino-2-phenylindole (blue) and AF647 anti-human IgG (red). Images were acquired using the Opera Phenix system (PerkinElmer) at 63× magnification and analyzed in Columbus (PerkinElmer).

In the opsonophagocytosis assays, MrkA-targeting mAbs had some effect at enhancing killing and macrophage clearance; however, in both assays, the mAbs were less active than the O-antigen targeting positive control. To explore this observation, we next examined antibody binding to *K. pneumoniae* 43816 using HCI. Confocal images revealed punctate staining of appendages on the bacteria, and in line with previously published work (41) (Fig. 5a and b). A heterogenous bacterial population was observed: some bacteria were highly decorated with MrkA, some were partially decorated, and in others, MrkA decoration was absent (Fig. 5a). We next quantified the percentage of the bacterial population positive for anti-MrkA binding using an anti-human Alexa Fluorophore (AF) 647-labeled detection mAb. We observed that when probed with MrkA-targeting mAbs, around 70% of bacteria were positive for AF647 binding, compared to 80% for the positive control mAb, which binds to O-antigen (Fig. 5c). Significant variation between replicates was observed with the anti-MrkA mAbs (Fig. 5a and b), and this variation was absent in the positive control mAb.

**Fig 6:**
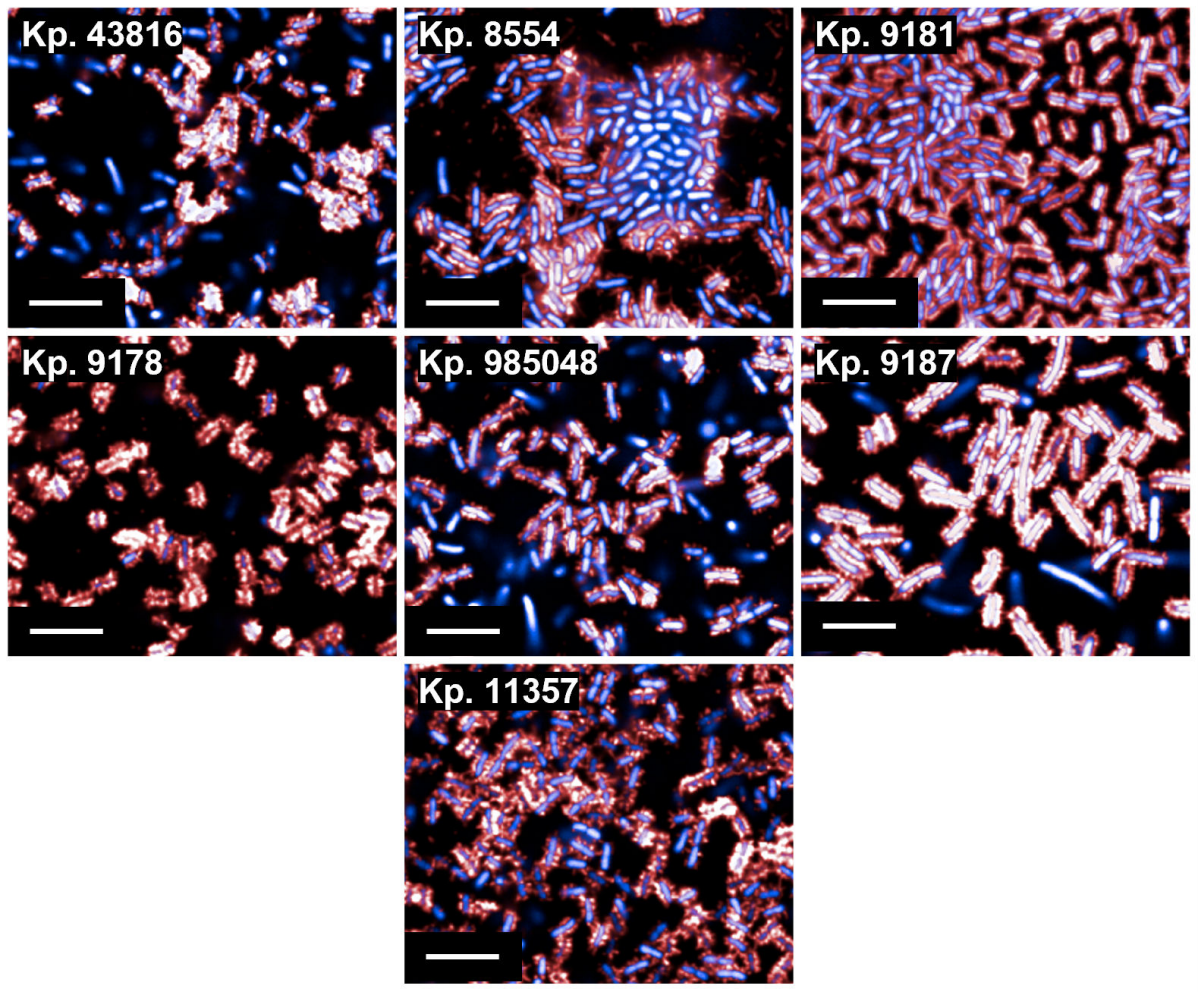
B07 binding to seven strains of *K. pneumoniae* by HCI. Fixed bacteria were treated with B07 (1 µg/mL) and then stained with 4′,6-diamidino-2-phenylindole (blue) + AF647 anti-Human IgG (red). Images were acquired using the Opera Phenix system (PerkinElmer) at 63× magnification and analyzed in Columbus (PerkinElmer). Scale bar represents 10 µm.

With the same panel of clinical isolates used in the cross-reactivity phage ELISA, we explored binding by HCI. It was noted that in some strains, nearly all the bacterial population was heavily decorated in fimbriae, while in others, sub-populations of decorated/non-decorated bacteria existed (Fig. 6). We observed that clinical isolates were more decorated in fimbriae than the 43816 “laboratory” strain.

## DISCUSSION

In this study, we report the discovery and characterization of mAbs-targeting MrkA, the type 3 fimbrial subunit found in *K. pneumoniae,* and other Enterobacteriacea. These mAbs were isolated in an extensive phage display campaign against live bacteria lacking bulky surface polysaccharides. HCI was used to investigate binding of the anti-MrkA mAbs, revealing heterogeneity in the surface expression of T3F.

In devising the phage display campaign, we anticipated that abundant surface polysaccharides including CPS and LPS could interfere with the phage display process, as such we utilized an acapsular mutant and an acapsular/LPS-deficient mutant alongside the WT. However, we were surprised to find that carbohydrate-targeting mAbs were rare in the antibody populations, even in outputs that enriched for WT bacteria at all three rounds of selection. Carbohydrate-targeting mAbs have been shown to have a 1 × 10^3^–1 × 10^5^-fold lower affinity than protein- or peptide-binding antibodies (46). The lower affinity of anti-carbohydrate mAbs is compensated for by their expression as the decavalent format IgM and subsequent class switching toward the IgG2 class which can self-associate via antibody constant regions to form a multivalent system (40). This strategy of multivalency allows for the recognition of densely displayed antigens such as the carbohydrates found on bacterial cell surfaces. In contrast to the high valency of the IgM and IgG2 formats, during phage display, the M13 bacteriophage can display a maximum five copies of scFv per phage particle; however, a typical phage particle will only display one copy of the scFv. It is possible that one copy does not provide sufficient valency/avidity required for the scFv-carbohydrate complex, which could explain why fewer carbohydrate-targeting scFvs were isolated.

Using HCI, under the conditions tested, we observed some bacteria completely lacking fimbriae, while others were heavily decorated in T3F. This could be consistent with the fact that heterogenous expression of virulence factors is an important survival tactic for bacteria to adapt to changing environments within the host. For example, type I fimbriae expression in *Escherichia coli* (47) is well characterized and is known to be phase variable. It should also be noted that T3F decoration was observed in bacteria grown in TY broth, a media in which fimbrial expression may not be advantageous or needed, which may further account for the heterogenous expression of T3F.

Similarly, *K. pneumoniae* is known to modulate the production of CPS (48). It is possible that T3F on a bacterium highly expressing CPS may be shielded, thereby reducing the ability of anti-MrkA mAbs to bind and could explain the observed heterogenous T3F expression. Future work should aim to study *mrkA* expression in bacteria grown under different conditions as well as in bacteria taken directly from a lung infection.

Treatment with anti-MrkA antibodies promoted 45% killing in a luminescence-based readout of OPK activity, which is in line with the activity seen in previous reports of anti-MrkA mAbs (43). When HCI was used to measure macrophage-associated bacteria, the anti-MrkA mAbs promoted a 30% reduction in *K. pneumoniae* spot intensity at the same timepoint. In comparison, the anti-LPS mAb promoted 95% killing and an 80% reduction in *K. pneumoniae* spot intensity. Interestingly, at the 7-hour timepoint, the anti-MrkA mAbs were no longer promoting a reduction in *K. pneumoniae* spot intensity. The heterogeneity of T3F expression observed within the bacterial population could account for both the reduced efficacy of anti-MrkA mAbs compared to anti-LPS mAbs and the change in efficacy from 5 to 7 hours. It is possible that the reduction in macrophage-associated *K. pneumoniae* is due to the clearance of bacteria with T3F surface expression and that the remaining bacterial population then proliferated, leading to the increase in *K. pneumoniae* spot intensity seen at 7 hours; future work should aim to explore this further.

It is intriguing that antibodies targeting MrkA have been identified in two independent campaigns (40, 41, 43) that utilized different approaches for lead generation (hybridoma and phage display were both used previously, and phage display was used here). Anti-MrkA mAbs identified previously showed potent *in vivo* protection in a prophylactic murine model, but when dosed therapeutically, protection was modest and only at a high dosing regimen (40). Our study has identified heterogeneity in MrkA expression within the bacterial population and a difference between strains, with clinical isolates seemingly expressing more MrkA (i.e., T3F) than lab-adapted strains. Therefore, we hypothesize that the reduced therapeutic efficacy reported could be due to the heterogeneity in MrkA expression as well as the strain tested. We contend that future work should aim to explore the potential for MrkA-targeting mAbs to prevent/clear infection using a panel of isolates (i.e., with the potential for different MrkA expression) as well as explore different models of infection (e.g., GI, bladder, and systemic).

MrkA is reported to be involved in biofilm formation, particularly on abiotic surfaces, including catheters, urogenital adhesion, and establishment of infection (26, 43). MrkA is an attractive antibody therapeutic target due to (i) its general accessibility as an extracellular target, (ii) its high degree of sequence conservation among different isolates (*K. pneumoniae* and *K. oxytoca* exhibit 95% amino acid sequence identity) and MrkA among Enterobacteriaceae (including *Salmonella*, *E. coli*, *Shigella*, and *Citrobacter*) is >90%, except for *Enterobacter cloacae*, which is divergent, (iii) anti-MrkA mAbs prevent *K. pneumoniae* association with lung epithelial cells and significantly block biofilm formation on abiotic surfaces, and (iv) anti-MrkA antibodies possess protective activities *in vivo* (26, 43). While further experiments are required, it is possible that targeting MrkA may be an effective strategy to reduce urinary tract infections (UTI) and catheter-associated UTI, caused by *K. pneumoniae* and other bacterial species expressing MrkA.

In summary, in this study, we have explored existing target-independent screening outputs and identified and characterized anti-MrkA mAbs that were found to be highly cross-reactive, binding to all *K. pneumoniae* strains tested from a diverse panel of clinical isolates, representing different O-serotypes, and were active in an OPK assay at pM concentrations. The T3F protein MrkA is important for biofilm formation; thus, our data support further exploration of the use of anti-MrkA antibodies for preventing and/or controlling *K. pneumoniae* in biofilms and possibly certain stages of the infection process.

## MATERIALS AND METHODS

### Bacterial strains and media

Bacteria used in this work were stored as 25% glycerol stocks at *−*80°C and cultured in tryptone/yeast extract broth. *K. pneumoniae* strains were purchased from the American Type Culture Collection, National Collection of Type Cultures, or International Health Management Associates. Strains used in this study are shown in Table S1. *K. pneumoniae* cultures were grown overnight at 37°C with 280 revolutions per minute (rpm) shaking. For use as the phagemid recipient in phage display, *E. coli* strain TG1 was grown at 37°C with 300 rpm shaking to an OD_600nm_ of 0.5. Broth was supplemented with ampicillin (100 µg/mL), kanamycin (50 µg/mL), and 2% (wt/vol) glucose, when necessary.

### Phage display on whole *K. pneumoniae* bacteria

Whole cell selections using live *K. pneumoniae* 43816 (WT), *K. pneumoniae* ∆*cpsB* (mutant), and *K. pneumoniae* ∆*cpsBwaaL* (double mutant) were performed as described previously (41). Briefly, 1 *×* 10^9^ cfu *K*. *pneumoniae* and 5 *×* 10^10^ phage particles from two combined naïve human scFv libraries (49, 50) were blocked in phosphate-buffered saline (PBS) supplemented with 3% non-fat dried milk and co-incubated. Unbound phage were removed by washing, and bound phage were eluted and used to infect mid-log phase *E. coli* TG1 cells for subsequent phage amplification. scFv sequences were analyzed to determine output diversity. Following three rounds of enrichment, scFvs were assessed for specific and non-specific binding to *K. pneumoniae* 43816, BSA, and *E. coli* TG1 by phage ELISA.

### Antibody engineering

Antibody engineering was performed as described previously (50). Briefly, scFvs were converted to scFv-Fc by ligating scFv sequences into a pOE-Fc vector containing the antibody Fc and expressed in 3 mL cultures of G22 Chinese hamster ovary (CHO) cells. scFvs were converted to IgG by cloning *V*_*H*_ and *V*_*L*_ DNA sequences into human IgG1 and human Lambda expression vectors, pEU 1.21 and pEU 4.4, respectively. IgG were expressed in 20 mL cultures of CHO cells for 6 days at 34°C. Purification of scFv-Fc and IgG was via protein A affinity chromatography using ÄKTA systems.

### Maximum likelihood phylogenetic tree construction

A maximum likelihood phylogenetic tree of a panel of 31 *K*. *pneumoniae* strains was constructed using multi-locus sequence typing (MLST) of housekeeping genes. MLST genes were extracted from the whole-genome fasta files using MLST-check (51). The genes were manually aligned, and maximum likelihood phylogenetic trees were estimated from the data using FastTree under the generalized time-reversible model of sequence evolution (52, 53). Support for individual nodes was estimated using Shimodaira-Hasegawa tests on the three alternate topologies around that split (54). Strong support for different topologies between the MLST genes indicates the presence of recombination. The genes *phoE* and *pgi* gave strong support for different phylogenetic groupings compared to the other MLST genes, so these were eliminated from further analysis. The remaining genes were concatenated together and tested for recombination events using small sample Akaike Information Criterion single breakpoint analysis (55). One recombination breakpoint was detected at base 1841; therefore, the concatenated alignment was ended at this point. The single breakpoint detection analysis was re-run, and no recombination was detected using small sample Akaike Information Criterion. The final phylogenetic tree was produced on this concatenated, recombination-free alignment using FastTree (53).

### Enzyme-linked immunosorbent assay

Whole-bacteria ELISA was performed as described previously (56). Briefly, plates were coated overnight at 4°C with 5 × 10^7^ cfu/well *K. pneumoniae* 43816 and then washed three times in PBS. scFv-expressing phage cultures and plates were blocked with 3% milk for 1 hour prior to a 1-hour co-incubation. Plates were washed three times with PBS supplemented with 0.1% Tween 20 (PBS-T), and then anti-M13 horseradish peroxidase (HRP) conjugate was added for 1 hour. Following three washes with PBS-T, binding was visualized with 5′ tetramethylbenzidine substrate (ThermoScientific), and the color change reaction was stopped with 0.5N H_2_SO_4_. Absorbance at 450 nm was measured using a plate reader (Envision).

For the protein vs carbohydrate phage ELISA, scFvs were tested for binding to *K. pneumoniae* 43816 bacteria, *K. pneumoniae* 43816 Δ*cpsBwaaL* bacteria, *K. pneumoniae* 43816 lysate, and *K. pneumoniae* 43816 proteinase-K-digested lysate. Bacterial lysates were prepared with B-PER bacterial protein extraction reagent (ThermoScientific). For protein degradation, lysates were treated with proteinase-K (Roche) at 55°C for 1 hour and then incubated at 70°C for 10 minutes to inactivate proteinase-K. Lysates were diluted 1 in 10 prior to coating overnight at 4°C. scFvs that bound to both lysates were assigned as carbohydrate binders, while scFvs that only bound non-digested lysate were assigned as protein binders. scFvs that did not bind to either lysate but bound to whole bacteria were assigned as “unknown.” For the ELISA using recombinant MrkA, mAbs at 1 µg/mL were tested for binding to MrkA at 1 µg/mL. Recombinant MrkA was expressed in *E. coli* using the *mrkA*-coding sequence from the reference strain *K. pneumoniae* MG78578, the production of which is as described previously (43). Plates were coated overnight at 4°C. Binding was detected with Goat anti-Human IgG (Fc specific) HRP conjugate (Invitrogen). For all ELISAs, Nunc maxisorp 96-well plates were used (BioLegend).

### Opsonophagocytic killing assay

OPK assays were performed as described previously (41). Briefly, log-phase cultures of luminescent *K. pneumoniae* were diluted to approximately 3.0 *×* 10^5^ cfu/mL in OPK buffer [RPMI 1640 medium without phenol red (Gibco) + 1% BSA (Sigma)]. Baby rabbit serum (Cedarlane) was diluted 1 in 10 in OPK buffer and incubated with *K. pneumoniae* for 1 hour to clear pre-existing antibodies. Test antibodies were serially diluted in OPK buffer. Bacteria, complement, and antibodies were added to 96-well white, clear bottom microplates (Corning) containing human monocyte-derived macrophages at 3.0 *×* 10^4^ cells/well. Plates were sealed with Breathe-Easy sealing membranes (Merck) and incubated at 37°C for 5 hours with 5% CO_2_. An Envision multilabel plate reader (Envision) was used to read total luminescence units, and OPK activity was calculated as a percentage of wells containing no IgG. Note: OPK assays were performed alongside testing mAbs reported in a previous publication by Berry et al. (41); the control images and data shown in Fig. 4 correspond to Nip223 (nIgG) and B39 (pIgG) in Berry et al. (41).

### High-content imaging

For HCI intracellular clearance studies, MDMs were seeded at 3.0 *×* 10^4^ cells/well in tissue-culture-treated 96-well optical bottom plates (Nunc). Assays were prepared as described for the OPK assay, in the absence of complement. At each timepoint, cells were fixed in 4% paraformaldehyde (PFA), then washed three times with PBS, and stained with cell mask orange (CMO; 1/25,000) (Invitrogen), Hoechst (1/10,000) (ThermoScientific), and 0.3% Triton, prepared in HBSS supplemented with 5% donkey serum (Jackson Laboratory). Cells were then washed three times in PBS and stained with rabbit polyclonal anti-*K*. *pneumoniae* 43816 antibody for 30 minutes. Following one wash in PBS, cells were stained with AF488 anti-rabbit IgG (Affinipure #111–545-003) for 30 minutes, before a final three washes in PBS. An Opera system (PerkinElmer) was used to image 15 fields per well at 20× magnification. For HCI binding characterization studies, 5.0 *×* 10^5^ *K. pneumoniae* bacteria were added to wells of a 96-well CellCarrier Ultra microplate (PerkinElmer) and incubated for 2 hours at 37°C. After fixing with 4% PFA for 10 minutes, bacteria were treated for 1 hour with primary antibodies at 1 µg/mL [a variety of concentrations were tested, and 1 µg/mL was found to be optimal (Fig. S3)]. Bacteria were then stained with 4′,6-diamidino-2-phenylindole (DAPI) (2 µg/mL) and AF647 anti-human IgG (Invitrogen #A-21445) (1 µg/mL). An Opera Phenix system (PerkinElmer) was used for image acquisition at 63× magnification.

### HCI analysis

HCI analysis was performed in Columbus (PerkinElmer). For quantification of MDM-associated *K. pneumoniae* spot intensity, the macrophage cytoplasm image area was first defined using CMO signal. *K. pneumoniae* spots within the macrophage cytoplasm image area were then defined using AF488 signal, and the total AF488 intensity sum was calculated, excluding spots with a mean AF488 intensity <5 to eliminate non-specific background signal. For quantification of the proportion of bacteria that were positive for binding, the number of bacteria with a shell region AF647 intensity >3,000 was divided by the total number of bacteria. For quantification of mAb binding to *K. pneumoniae* 43816, the total AF647 intensity per well was calculated using a cutoff of mean >3,000 to eliminate non-specific background signal. This signal was normalized to the density of bacteria per well by dividing the AF647 signal by DAPI area (px^2^).

### Statistical analysis

All statistical analyses were performed in GraphPad Prism, version 8.

## Data Availability

The data that support the findings of this study are available from the corresponding author upon reasonable request. Genome sequences are available to donwload here: https://doi.org/10.17863/CAM.88687.

## SUPPLEMENTAL MATERIAL

Figure S1 spectrum.00400-24-s0001.tif Maximum likelihood phylogenetic tree of a panel of *K. pneumoniae* strains.

Figure S2 spectrum.00400-24-s0002.tif Opsonophagocytic killing of *K. pneumoniae* 43816 by macrophages in the presence of MrkA-targeting mAbs.

Figure S3 spectrum.00400-24-s0003.tif Binding of MrkA-targeting mAbs in a dilution series to *K. pneumoniae*.

Supplemental material spectrum.00400-24-s0004.docx Figure legends.

Table S1 spectrum.00400-24-s0005.docx Bacterial strains used in this study.

ASM does not own the copyrights to Supplemental Material that may be linked to, or accessed through, an article. The authors have granted ASM a non-exclusive, world-wide license to publish the Supplemental Material files. Please contact the corresponding author directly for reuse.

## References

[1] Choi M, Tennant SM, Simon R, Cross AS. 2019. Progress towards the development of Klebsiella vaccines. Expert Rev Vaccines 18:681–691. doi:10.1080/14760584.2019.1635460 31250679 PMC6656602

[2] Podschun R, Ullmann U. 1998. Klebsiella spp. as nosocomial pathogens: epidemiology, taxonomy, typing methods, and pathogenicity factors. Clin Microbiol Rev 11:589–603. doi:10.1128/CMR.11.4.589 9767057 PMC88898

[3] Rock C, Thom KA, Masnick M, Johnson JK, Harris AD, Morgan DJ. 2014. Frequency of Klebsiella pneumoniae carbapenemase (KPC)-producing and non-KPC-producing Klebsiella species contamination of healthcare workers and the environment. Infect Control Hosp Epidemiol 35:426–429. doi:10.1086/675598 24602950 PMC4030386

[4] Nguyen GT, Shaban L, Mack M, Swanson KD, Bunnell SC, Sykes DB, Mecsas J. 2020. SKAP2 is required for defense against K. pneumoniae infection and neutrophil respiratory burst. Elife 9:e56656. doi:10.7554/eLife.56656 32352382 PMC7250567

[5] Khaertynov KS, Anokhin VA, Rizvanov AA, Davidyuk YN, Semyenova DR, Lubin SA, Skvortsova NN. 2018. Virulence factors and antibiotic resistance of Klebsiella pneumoniae strains isolated from neonates with sepsis. Front Med (Lausanne) 5:225. doi:10.3389/fmed.2018.00225 30155466 PMC6102385

[6] Ghotaslou R, Ghorashi Z, Nahaei MR. 2007. Klebsiella pneumoniae in neonatal sepsis: a 3-year-study in the pediatric hospital of Tabriz, Iran. Jpn J Infect Dis 60:126–128. doi:10.7883/yoken.JJID.2007.126 17515647

[7] Du N, Liu S, Niu M, Duan Y, Zhang S, Yao J, Mao J, Chen R, Du Y. 2017. Transmission and characterization of bla_NDM-1_ in Enterobacter cloacae at a teaching hospital in Yunnan, China. Ann Clin Microbiol Antimicrob 16:58. doi:10.1186/s12941-017-0232-y 28830556 PMC5568220

[8] Ramos-Castañeda JA, Ruano-Ravina A, Barbosa-Lorenzo R, Paillier-Gonzalez JE, Saldaña-Campos JC, Salinas DF, Lemos-Luengas EV. 2018. Mortality due to KPC carbapenemase-producing Klebsiella pneumoniae infections: systematic review and meta-analysis: mortality due to KPC Klebsiella pneumoniae infections. J Infect 76:438–448. doi:10.1016/j.jinf.2018.02.007 29477802

[9] Lam MMC, Wick RR, Watts SC, Cerdeira LT, Wyres KL, Holt KE. 2021. A genomic surveillance framework and genotyping tool for Klebsiella pneumoniae and its related species complex. Nat Commun 12:4188. doi:10.1038/s41467-021-24448-3 34234121 PMC8263825

[10] Argimón S, David S, Underwood A, Abrudan M, Wheeler NE, Kekre M, Abudahab K, Yeats CA, Goater R, Taylor B, Harste H, Muddyman D, Feil EJ, Brisse S, Holt K, Donado-Godoy P, Ravikumar KL, Okeke IN, Carlos C, Aanensen DM, NIHR Global Health Research Unit on Genomic Surveillance of Antimicrobial Resistance. 2021. Rapid genomic characterization and global surveillance of Klebsiella using pathogenwatch. Clin Infect Dis 73:S325–S335. doi:10.1093/cid/ciab784 34850838 PMC8634497

[11] Foster-Nyarko E, Cottingham H, Wick RR, Judd LM, Lam MMC, Wyres KL, Stanton TD, Tsang KK, David S, Aanensen DM, Brisse S, Holt KE. 2023. Nanopore-only assemblies for genomic surveillance of the global priority drug-resistant pathogen, Klebsiella pneumoniae. Microb Genom 9:mgen000936. doi:10.1099/mgen.0.000936 36752781 PMC9997738

[12] Chang D, Sharma L, Dela Cruz CS, Zhang D. 2021. Clinical epidemiology, risk factors, and control strategies of Klebsiella pneumoniae infection. Front Microbiol 12:750662. doi:10.3389/fmicb.2021.750662 34992583 PMC8724557

[13] Karampatakis T, Tsergouli K, Behzadi P. 2023. Carbapenem-resistant Klebsiella pneumoniae: virulence factors, molecular epidemiology and latest updates in treatment options. Antibiotics (Basel) 12:234. doi:10.3390/antibiotics12020234 36830145 PMC9952820

[14] Tilahun M, Kassa Y, Gedefie A, Ashagire M. 2021. Emerging carbapenem-resistant Enterobacteriaceae infection, its epidemiology and novel treatment options: a review. Infect Drug Resist 14:4363–4374. doi:10.2147/IDR.S337611 34707380 PMC8544126

[15] Arcari G, Carattoli A. 2023. Global spread and evolutionary convergence of multidrug-resistant and hypervirulent Klebsiella pneumoniae high-risk clones. Pathog Glob Health 117:328–341. doi:10.1080/20477724.2022.2121362 36089853 PMC10177687

[16] Lipworth S, Vihta KD, Chau K, Barker L, George S, Kavanagh J, Davies T, Vaughan A, Andersson M, Jeffery K, Oakley S, Morgan M, Hopkins S, Peto TEA, Crook DW, Walker AS, Stoesser N. 2021. Ten-year longitudinal molecular epidemiology study of Escherichia coli and Klebsiella species bloodstream infections in Oxfordshire, UK. Genome Med 13:144. doi:10.1186/s13073-021-00947-2 34479643 PMC8414751

[17] Bengoechea JA, Sa Pessoa J. 2019. Klebsiella pneumoniae infection biology: living to counteract host defences. FEMS Microbiol Rev 43:123–144. doi:10.1093/femsre/fuy043 30452654 PMC6435446

[18] Dobson CL, Devine PWA, Phillips JJ, Higazi DR, Lloyd C, Popovic B, Arnold J, Buchanan A, Lewis A, Goodman J, et al.. 2016. Engineering the surface properties of a human monoclonal antibody prevents self-association and rapid clearance in vivo. Sci Rep 6:38644. doi:10.1038/srep38644 27995962 PMC5171805

[19] Nagy E, Nagy G, Power CA, Badarau A, Szijártó V. 2017. Anti-bacterial monoclonal antibodies. Adv Exp Med Biol 1053:119–153. doi:10.1007/978-3-319-72077-7_7 29549638

[20] Vij R, Lin Z, Chiang N, Vernes J-M, Storek KM, Park S, Chan J, Meng YG, Comps-Agrar L, Luan P, Lee S, Schneider K, Bevers J, Zilberleyb I, Tam C, Koth CM, Xu M, Gill A, Auerbach MR, Smith PA, Rutherford ST, Nakamura G, Seshasayee D, Payandeh J, Koerber JT. 2018. A targeted boost-and-sort immunization strategy using Escherichia coli BamA identifies rare growth inhibitory antibodies. Sci Rep 8:7136. doi:10.1038/s41598-018-25609-z 29740124 PMC5940829

[21] Storek KM, Auerbach MR, Shi H, Garcia NK, Sun D, Nickerson NN, Vij R, Lin Z, Chiang N, Schneider K, Wecksler AT, Skippington E, Nakamura G, Seshasayee D, Koerber JT, Payandeh J, Smith PA, Rutherford ST. 2018. Monoclonal antibody targeting the β-barrel assembly machine of Escherichia coli is bactericidal. Proc Natl Acad Sci U S A 115:3692–3697. doi:10.1073/pnas.1800043115 29555747 PMC5889671

[22] Hua L, Cohen TS, Shi Y, Datta V, Hilliard JJ, Tkaczyk C, Suzich J, Stover CK, Sellman BR. 2015. MEDI4893* promotes survival and extends the antibiotic treatment window in a Staphylococcus aureus immunocompromised pneumonia model. Antimicrob Agents Chemother 59:4526–4532. doi:10.1128/AAC.00510-15 25987629 PMC4505239

[23] DiGiandomenico A, Keller AE, Gao C, Rainey GJ, Warrener P, Camara MM, Bonnell J, Fleming R, Bezabeh B, Dimasi N, Sellman BR, Hilliard J, Guenther CM, Datta V, Zhao W, Gao C, Yu XQ, Suzich JA, Stover CK. 2014. A multifunctional bispecific antibody protects against Pseudomonas aeruginosa. Sci Transl Med 6:262ra155. doi:10.1126/scitranslmed.3009655 25391481

[24] François B, Mercier E, Gonzalez C, Asehnoune K, Nseir S, Fiancette M, Desachy A, Plantefève G, Meziani F, de Lame P-A, Laterre P-F, MASTER1 study group. 2018. Safety and tolerability of a single administration of AR-301, a human monoclonal antibody, in ICU patients with severe pneumonia caused by Staphylococcus aureus: first-in-human trial. Intensive Care Med 44:1787–1796. doi:10.1007/s00134-018-5229-2 30343314

[25] Zurawski DV, McLendon MK. 2020. Monoclonal antibodies as an antibacterial approach against bacterial pathogens. Antibiotics (Basel) 9:155. doi:10.3390/antibiotics9040155 32244733 PMC7235762

[26] Motley MP, Fries BC. 2017. A new take on an old remedy: generating antibodies against multidrug-resistant Gram-negative bacteria in a postantibiotic world. mSphere 2:e00397-17. doi:10.1128/mSphere.00397-17 28989972 PMC5628292

[27] Dufner P, Jermutus L, Minter RR. 2006. Harnessing phage and ribosome display for antibody optimisation. Trends Biotechnol 24:523–529. doi:10.1016/j.tibtech.2006.09.004 17000017

[28] Wang Q, Chen Y, Park J, Liu X, Hu Y, Wang T, McFarland K, Betenbaugh MJ. 2019. Design and production of bispecific antibodies. Antibodies (Basel) 8:43. doi:10.3390/antib8030043 31544849 PMC6783844

[29] Cohen TS, Pelletier M, Cheng L, Pennini ME, Bonnell J, Cvitkovic R, Chang C-S, Xiao X, Cameroni E, Corti D, Semenova E, Warrener P, Sellman BR, Suzich J, Wang Q, Stover CK. 2017. Anti-LPS antibodies protect against Klebsiella pneumoniae by empowering neutrophil-mediated clearance without neutralizing TLR4. JCI Insight 2:e92774. doi:10.1172/jci.insight.92774 28469079 PMC5414560

[30] Pennini ME, De Marco A, Pelletier M, Bonnell J, Cvitkovic R, Beltramello M, Cameroni E, Bianchi S, Zatta F, Zhao W, Xiao X, Camara MM, DiGiandomenico A, Semenova E, Lanzavecchia A, Warrener P, Suzich J, Wang Q, Corti D, Stover CK. 2017. Immune stealth-driven O2 serotype prevalence and potential for therapeutic antibodies against multidrug resistant Klebsiella pneumoniae. Nat Commun 8:1991. doi:10.1038/s41467-017-02223-7 29222409 PMC5722860

[31] Szijártó V, Guachalla LM, Hartl K, Varga C, Badarau A, Mirkina I, Visram ZC, Stulik L, Power CA, Nagy E, Nagy G. 2017. Endotoxin neutralization by an O-antigen specific monoclonal antibody: a potential novel therapeutic approach against Klebsiella pneumoniae ST258. Virulence 8:1203–1215. doi:10.1080/21505594.2017.1279778 28103139 PMC5711440

[32] Diago-Navarro E, Motley MP, Ruiz-Peréz G, Yu W, Austin J, Seco BMS, Xiao G, Chikhalya A, Seeberger PH, Fries BC. 2018. Novel, broadly reactive anticapsular antibodies against carbapenem-resistant Klebsiella pneumoniae protect from infection. mBio 9:e00091-18. doi:10.1128/mBio.00091-18 29615497 PMC5885035

[33] Diago-Navarro E, Calatayud-Baselga I, Sun D, Khairallah C, Mann I, Ulacia-Hernando A, Sheridan B, Shi M, Fries BC. 2017. Antibody-based immunotherapy to treat and prevent infection with hypervirulent Klebsiella pneumoniae. Clin Vaccine Immunol 24:e00456-16. doi:10.1128/CVI.00456-16 27795303 PMC5216427

[34] Kobayashi SD, Porter AR, Freedman B, Pandey R, Chen L, Kreiswirth BN, DeLeo FR. 2018. Antibody-mediated killing of carbapenem-resistant ST258 Klebsiella pneumoniae by human neutrophils. mBio 9:e00297-18. doi:10.1128/mBio.00297-18 29535199 PMC5850326

[35] Guachalla LM, Stojkovic K, Hartl K, Kaszowska M, Kumar Y, Wahl B, Paprotka T, Nagy E, Lukasiewicz J, Nagy G, Szijártó V. 2017. Discovery of monoclonal antibodies cross-reactive to novel subserotypes of K. pneumoniae O3. Sci Rep 7:6635. doi:10.1038/s41598-017-06682-2 28747785 PMC5529442

[36] Trautmann M, Ruhnke M, Rukavina T, Held TK, Cross AS, Marre R, Whitfield C. 1997. O-antigen seroepidemiology of Klebsiella clinical isolates and implications for immunoprophylaxis of Klebsiella infections. Clin Diagn Lab Immunol 4:550–555. doi:10.1128/cdli.4.5.550-555.1997 9302204 PMC170594

[37] Aytenfisu AH, Simon R, MacKerell AD. 2019. Impact of branching on the conformational heterogeneity of the lipopolysaccharide from Klebsiella pneumoniae: implications for vaccine design. Carbohydr Res 475:39–47. doi:10.1016/j.carres.2019.02.003 30818097 PMC6529184

[38] Micoli F, Costantino P, Adamo R. 2018. Potential targets for next generation antimicrobial glycoconjugate vaccines. FEMS Microbiol Rev 42:388–423. doi:10.1093/femsre/fuy011 29547971 PMC5995208

[39] Follador R, Heinz E, Wyres KL, Ellington MJ, Kowarik M, Holt KE, Thomson NR. 2016. The diversity of Klebsiella pneumoniae surface polysaccharides. Microb Genom 2:e000073. doi:10.1099/mgen.0.000073 28348868 PMC5320592

[40] Wang Q, Chen Y, Cvitkovic R, Pennini ME, Chang CS, Pelletier M, Bonnell J, Koksal AC, Wu H, Dall’Acqua WF, Stover CK, Xiao X. 2017. Anti-MrkA monoclonal antibodies reveal distinct structural and antigenic features of MrkA. PLoS One 12:e0170529. doi:10.1371/journal.pone.0170529 28107434 PMC5249199

[41] Berry SK, Rust S, Caceres C, Irving L, Bartholdson Scott J, Tabor DE, Dougan G, Christie G, Warrener P, Minter R, Grant AJ. 2022. Phenotypic whole-cell screening identifies a protective carbohydrate epitope on Klebsiella pneumoniae. MAbs 14:2006123. doi:10.1080/19420862.2021.2006123 34923908 PMC8726669

[42] Rollenske T, Szijarto V, Lukasiewicz J, Guachalla LM, Stojkovic K, Hartl K, Stulik L, Kocher S, Lasitschka F, Al-Saeedi M, Schröder-Braunstein J, von Frankenberg M, Gaebelein G, Hoffmann P, Klein S, Heeg K, Nagy E, Nagy G, Wardemann H. 2018. Cross-specificity of protective human antibodies against Klebsiella pneumoniae LPS O-antigen. Nat Immunol 19:617–624. doi:10.1038/s41590-018-0106-2 29760533

[43] Wang Q, Chang C-S, Pennini M, Pelletier M, Rajan S, Zha J, Chen Y, Cvitkovic R, Sadowska A, Heidbrink Thompson J, Yu Lin H, Barnes A, Rickert K, Wilson S, Stover CK, Dall’Acqua WF, Chowdhury PS, Xiao X. 2016. Target-agnostic identification of functional monoclonal antibodies against Klebsiella pneumoniae multimeric MrkA fimbrial subunit. J Infect Dis 213:1800–1808. doi:10.1093/infdis/jiw021 26768253

[44] Amako K, Meno Y, Takade A. 1988. Fine structures of the capsules of Klebsiella pneumoniae and Escherichia coli K1. J Bacteriol 170:4960–4962. doi:10.1128/jb.170.10.4960-4962.1988 3049556 PMC211547

[45] Rukavina T, Tícac B, Susa M, Jendrike N, Jonjíc S, Lucin P, Marre R, Doríc M, Trautmann M. 1997. Protective effect of antilipopolysaccharide monoclonal antibody in experimental Klebsiella infection. Infect Immun 65:1754–1760. doi:10.1128/iai.65.5.1754-1760.1997 9125558 PMC175211

[46] Haji-Ghassemi O, Blackler RJ, Martin Young N, Evans SV. 2015. Antibody recognition of carbohydrate epitopes†. Glycobiology 25:920–952. doi:10.1093/glycob/cwv037 26033938

[47] Eisenstein BI. 1981. Phase variation of type 1 fimbriae in Escherichia coli is under transcriptional control. Science 214:337–339. doi:10.1126/science.6116279 6116279

[48] Dorman MJ, Feltwell T, Goulding DA, Parkhill J, Short FL. 2018. The capsule regulatory network of Klebsiella pneumoniae defined by density-TraDISort. mBio 9:e01863-18. doi:10.1128/mBio.01863-18 30459193 PMC6247091

[49] Vaughan TJ, Williams AJ, Pritchard K, Osbourn JK, Pope AR, Earnshaw JC, McCafferty J, Hodits RA, Wilton J, Johnson KS. 1996. Human antibodies with sub-nanomolar affinities isolated from a large non-immunized phage display library. Nat Biotechnol 14:309–314. doi:10.1038/nbt0396-309 9630891

[50] Lloyd C, Lowe D, Edwards B, Welsh F, Dilks T, Hardman C, Vaughan T. 2009. Modelling the human immune response: performance of a 1011 human antibody repertoire against a broad panel of therapeutically relevant antigens. Protein Eng Des Sel 22:159–168. doi:10.1093/protein/gzn058 18974080

[51] Page AJ, Taylor B, Keane JA. 2016. Multilocus sequence typing by blast from de novo assemblies against PubMLST. JOSS 1:118. doi:10.21105/joss.00118

[52] Tavaré S. 1986. Some probabilistic and statistical problems in the analysis of DNA sequences, p 57–86. In American mathematical society: lectures on mathematics in the life sciences. Vol. 17.

[53] Price MN, Dehal PS, Arkin AP. 2009. FastTree: computing large minimum evolution trees with profiles instead of a distance matrix. Mol Biol Evol 26:1641–1650. doi:10.1093/molbev/msp077 19377059 PMC2693737

[54] Price MN, Dehal PS, Arkin AP. 2010. FastTree 2--approximately maximum-likelihood trees for large alignments. PLoS One 5:e9490. doi:10.1371/journal.pone.0009490 20224823 PMC2835736

[55] Kosakovsky Pond SL, Posada D, Gravenor MB, Woelk CH, Frost SDW. 2006. Automated phylogenetic detection of recombination using a genetic algorithm. Mol Biol Evol 23:1891–1901. doi:10.1093/molbev/msl051 16818476

[56] DiGiandomenico A, Warrener P, Hamilton M, Guillard S, Ravn P, Minter R, Camara MM, Venkatraman V, Macgill RS, Lin J, Wang Q, Keller AE, Bonnell JC, Tomich M, Jermutus L, McCarthy MP, Melnick DA, Suzich JA, Stover CK. 2012. Identification of broadly protective human antibodies to Pseudomonas aeruginosa exopolysaccharide Psl by phenotypic screening. J Exp Med 209:1273–1287. doi:10.1084/jem.20120033 22734046 PMC3405507

