# Supplementary figures and images for: Characterization of mAbs against Klebsiella pneumoniae type 3 fimbriae isolated in a target-independent phage display campaign
Source: Microbiol Spectr. 2024 Jun 28;12(8):e00400-24. doi: 10.1128/spectrum.00400-24 (PMC11302298; doi:10.1128/spectrum.00400-24)

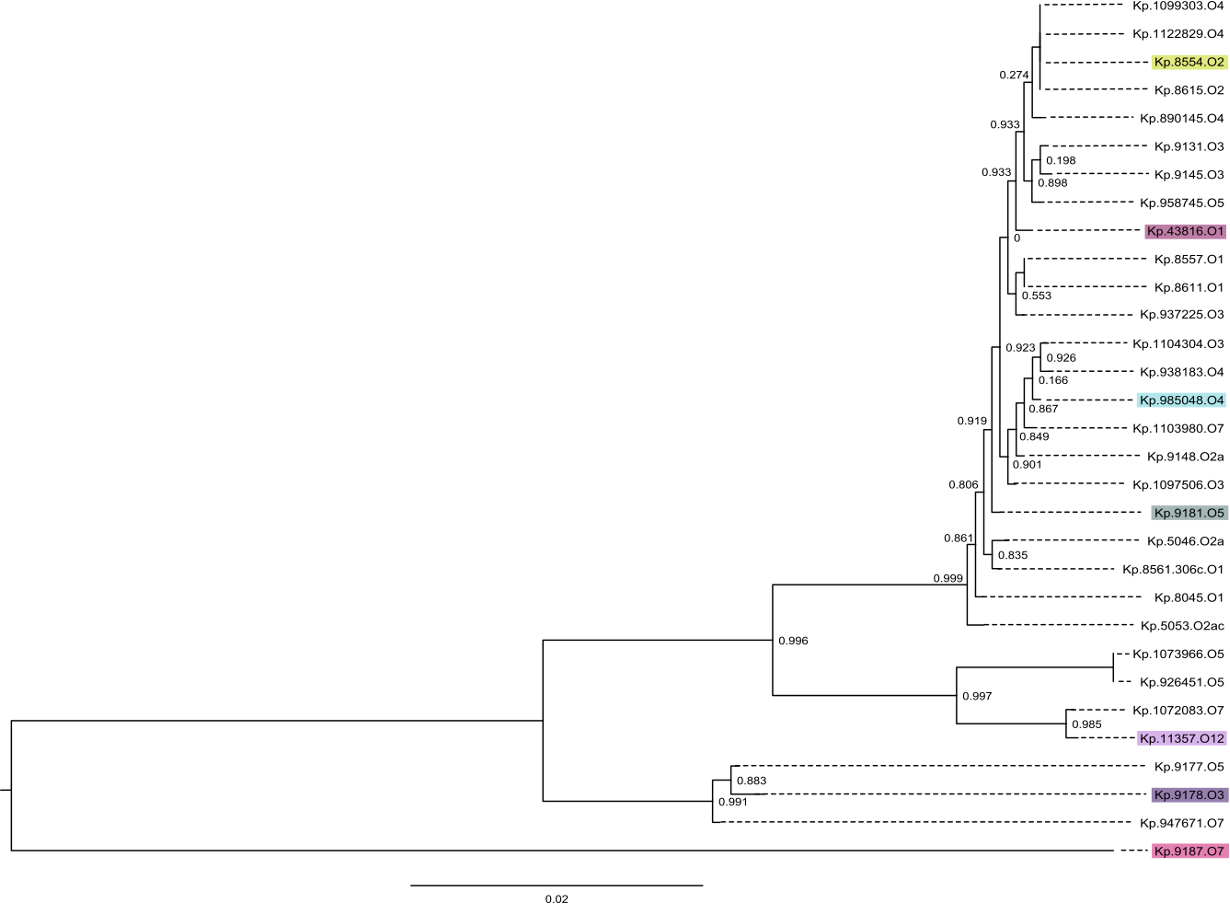

Supplement: Figure S1 — Maximum likelihood phylogenetic tree of a panel of K. pneumoniae strains. [file spectrum.00400-24-s0001.tif]

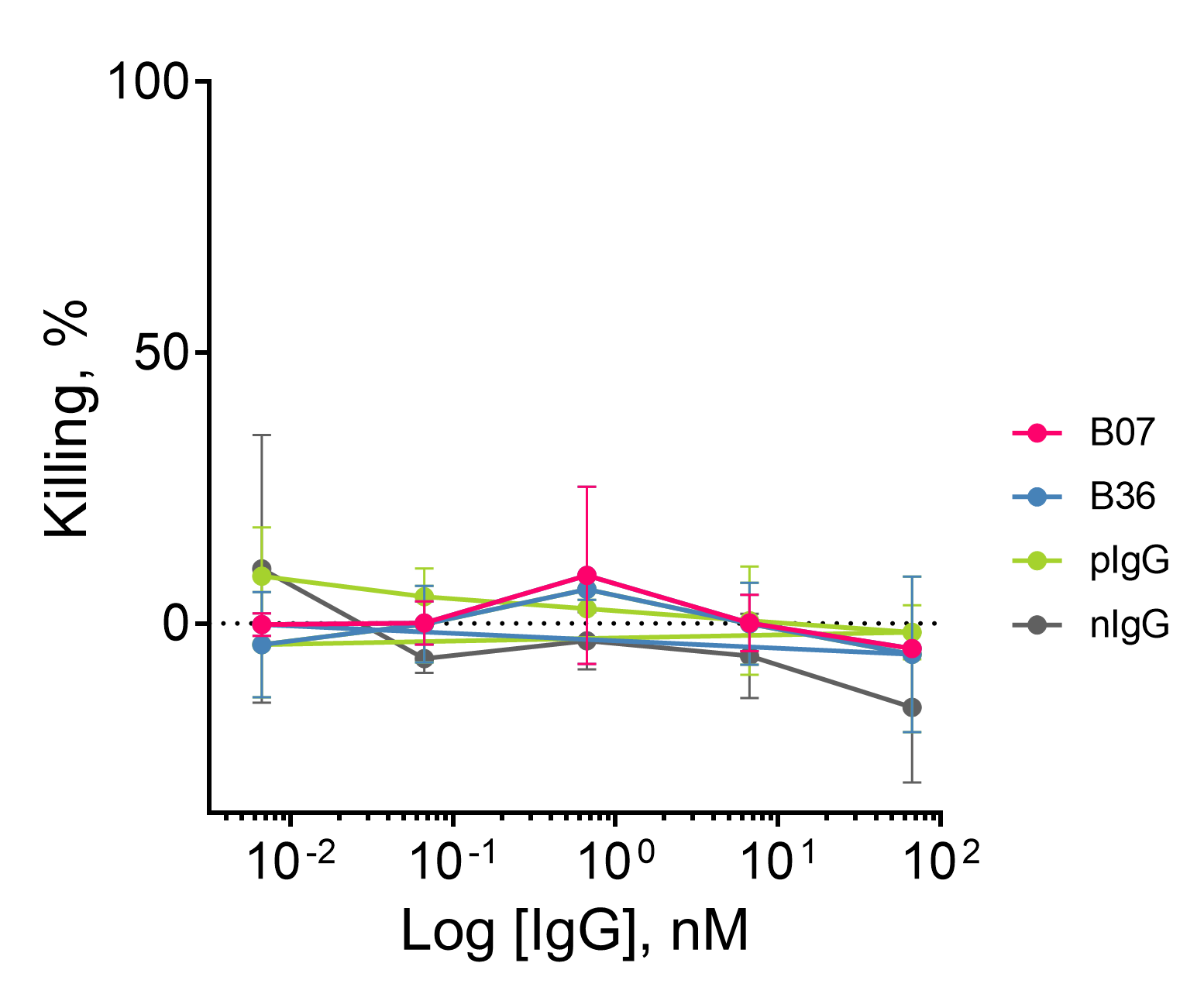

Supplement: Figure S2 — Opsonophagocytic killing of K. pneumoniae 43816 by macrophages in the presence of MrkA-targeting mAbs. [file spectrum.00400-24-s0002.tif]

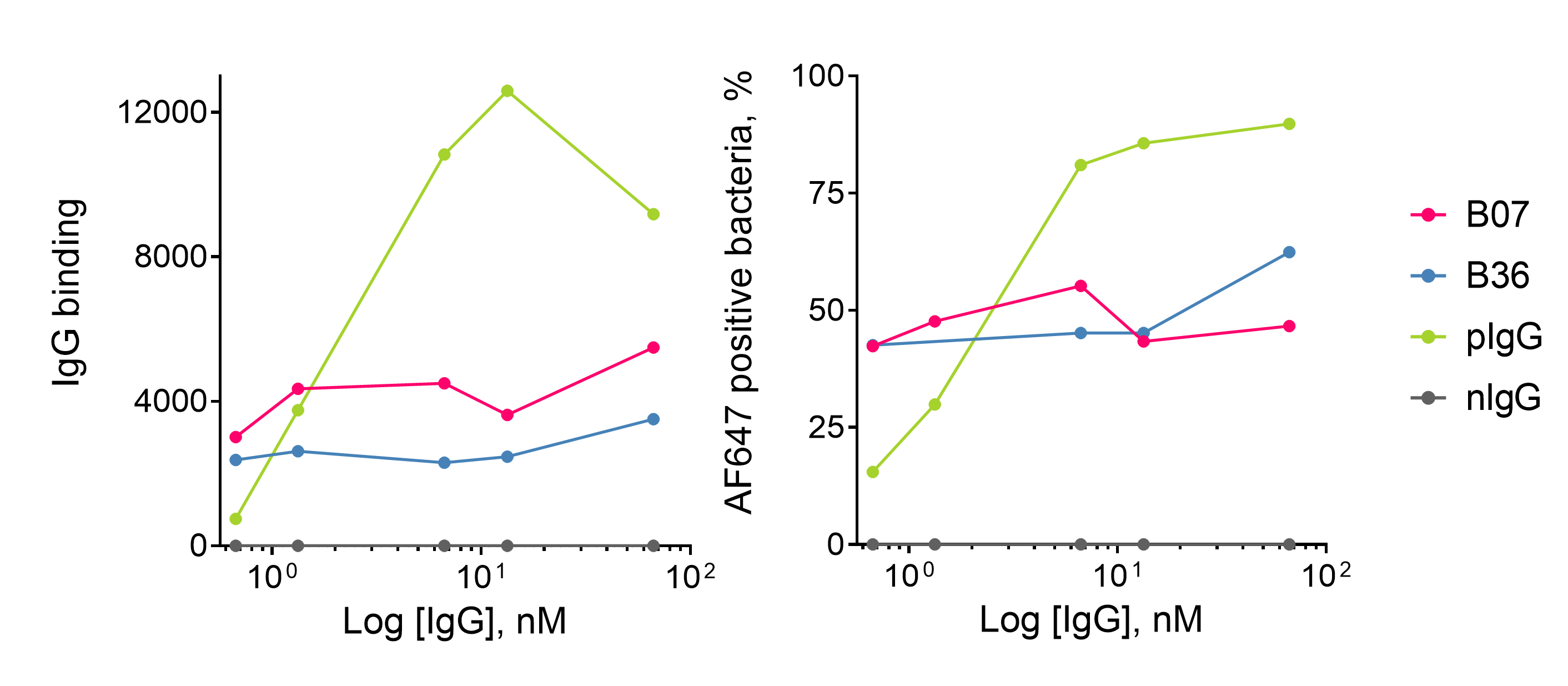

Supplement: Figure S3 — Binding of MrkA-targeting mAbs in a dilution series to K. pneumoniae. [file spectrum.00400-24-s0003.tif]
