# Supplementary material for: Characterization of mAbs against Klebsiella pneumoniae type 3 fimbriae isolated in a target-independent phage display campaign
Source: Microbiol Spectr. 2024 Jun 28;12(8):e00400-24. doi: 10.1128/spectrum.00400-24 (PMC11302298; doi:10.1128/spectrum.00400-24)
Supplement: Supplemental material — Figure legends. [file spectrum.00400-24-s0004.docx]

Supplemental Material – Figure Legends

**FIG S1** Maximum likelihood phylogenetic tree of a panel of *K. pneumoniae* strains. The tree was constructed using MLST of housekeeping genes. Strain names are appended with their O-antigen type. Strains selected for the cross-reactivity assay are highlighted in colour. Support for individual nodes is indicated. Scalebar indicates the number of substitutions per nucleotide.

**FIG S2** Opsonophagocytic killing of *K. pneumoniae* 43816 by macrophages in the presence of MrkA-targeting mAbs. *K. pneumoniae* 43816 *lux* bacteria, mAbs and complement were added to plates containing macrophages and incubated for 5 hours. Luminescence was measured using an Envision multilabel plate reader (PerkinElmer). pIgG = O-antigen binding mAb. nIgG = negative isotype control. Killing by test mAb or control mAb was calculated as a percentage of wells containing no mAb using the following calculation: (mAb treatment/no mAb)*100. Error bars represent 1 SD. N = 3 individual macrophage donors.

**FIG S3.** Binding of MrkA-targeting mAbs in a dilution series to *K. pneumoniae* 43816 by high-content imaging. Fixed bacteria were treated with mAbs at a range of concentrations then stained with DAPI (blue) and AF647 anti-human IgG (red). Images were acquired using the Opera Phenix system (PerkinElmer) at 63x magnification and analysed in Columbus (PerkinElmer). (**a**) Total IgG binding. IgG binding was calculated by dividing AF647 intensity sum by DAPI area. (**b**) Proportion of bacteria expressing MrkA when probed with B07, B36 or control mAbs. AF647 positive bacteria, % represents the percentage of bacteria with a shell region AF647 intensity > 3000 divided by the total number of bacteria.
