# Supplementary material for: Characterization of mAbs against Klebsiella pneumoniae type 3 fimbriae isolated in a target-independent phage display campaign
Source: Microbiol Spectr. 2024 Jun 28;12(8):e00400-24. doi: 10.1128/spectrum.00400-24 (PMC11302298; doi:10.1128/spectrum.00400-24)
Supplement: Table S1 — Bacterial strains used in this study. [file spectrum.00400-24-s0005.docx]

Supplemental Material – Table

**Table S1.** Bacterial strains used in this study.

| **Strain** | **Serotype*** | **Source** | **Additional information** |
| --- | --- | --- | --- |
| *Klebsiella pneumoniae* |  |  |  |
| 43816 | O1:K2 | ATCC |  |
| 43816 Δ*cpsB* | O1 | ^24^ | Capsule deficient |
| 43816 Δ*cpsBwaaL* | O ^-^ | ^30^ | Capsule/O-antigen deficient |
| 43816 *lux* | O1:K2 | ^30^ | Luminescent |
| 43816 Δ*cpsB* *lux* | O1 | ^24^ | Capsule deficient, luminescent |
| 8554 | O2 | Eurofin |  |
| 9178 | O3:K58 | NCTC | Sputum |
| 985048 | O4 | IHMA | Wound |
| 9181 | O5:K61 | NCTC | Sputum |
| 9187 | O7:K67 | NCTC |  |
| 11357 | O12:K80 | IHMA |  |
|  |  |  |  |
| *Escherichia coli* |  |  |  |
| TG1 |  | ^50^ | Amber suppressor |

*Capsule type indicated when known.
